# Anti-Oxidative Activity of Mytiloxanthin, a Metabolite of Fucoxanthin in Shellfish and Tunicates

**DOI:** 10.3390/md14050093

**Published:** 2016-05-11

**Authors:** Takashi Maoka, Azusa Nishino, Hiroyuki Yasui, Yumiko Yamano, Akimori Wada

**Affiliations:** 1 Research Institute for Production Development, 15 Shimogamo, Morimoto Cho, Sakyoku, Kyoto 606-0805, Japan; 2 Institute of Health Sciences, Ezaki Glico Co., Ltd., 4-6-5 Utajima, Nishiyodogawa-ku, Osaka 555-8502, Japan; nishino-azusa@gf.glico.co.jp; 3 Department of Analytical and Bioinorganic Chemistry, Division of Analytical and Physical Chemistry, Kyoto Pharmaceutical University, 5 Nakauchi-cho, Misasagi, Yamashina-ku, Kyoto 607-8414, Japan; yasui@mb.kyoto-phu.ac.jp; 4 Department of Organic Chemistry for Life Science, Kobe Pharmaceutical University, Motoyamakita-machi, Higashinada-ku, Kobe 658-8558, Japan; y-yamano@kobepharma-u.ac.jp (Y.Y.); a-wada@kobepharma-u.ac.jp (A.W.)

**Keywords:** mytiloxanthin, anti-oxidative activity, singlet oxygen, hydroxyl radical, lipid peroxidation

## Abstract

Anti-oxidative activities of mytiloxanthin, a metabolite of fucoxanthin in shellfish and tunicates, were investigated. Mytiloxanthin showed almost the same activities for quenching singlet oxygen and the inhibition of lipid peroxidation as those of astaxanthin, which is a well-known singlet oxygen quencher. Furthermore, mytiloxanthin showed excellent scavenging activity for hydroxyl radicals and this activity was markedly higher than that of astaxanthin.

## 1. Introduction

**Figure 1:**
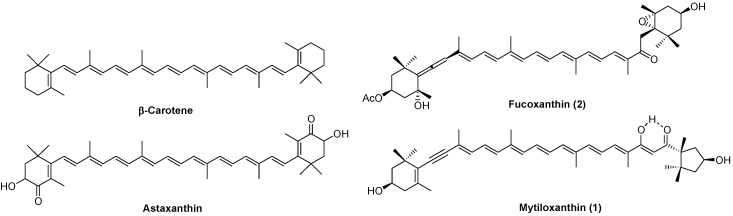
Structure of carotenoids used in this study.

Mytiloxanthin (**1**) is a carotenoid possessing a unique cyclopentyl enolic β-diketone group (Figure 1). It is distributed in several marine invertebrates such as shellfish and tunicates [1,2]. It was first isolated from the sea mussel *Mytilus californianus* by Sheer [3]. Its structure was determined to be 3,3′,8′-trihydroxy-7,8-didehydro-β,κ-caroten-6′-one through chemical and spectroscopic studies by Khare *et al.*, in 1973 [4]. Subsequently, Chopra *et al.* synthesized 9*Z*-(3*R*,3′*S*,5′*R*)-mytiloxanthin [5]. Its absolute configuration was determined to be (3*R*,3′*S*,5′*R*) using a modified Mosher’s method by Maoka and Fujiwara [6]. Total synthesis of all-*E*-(3*R*,3′*S*,5′*R*)-mytiloxanthin was achieved for the first time by Tode *et al.* [7], and it was recently improved by Yamano *et al.* [8].

**Figure 2:**
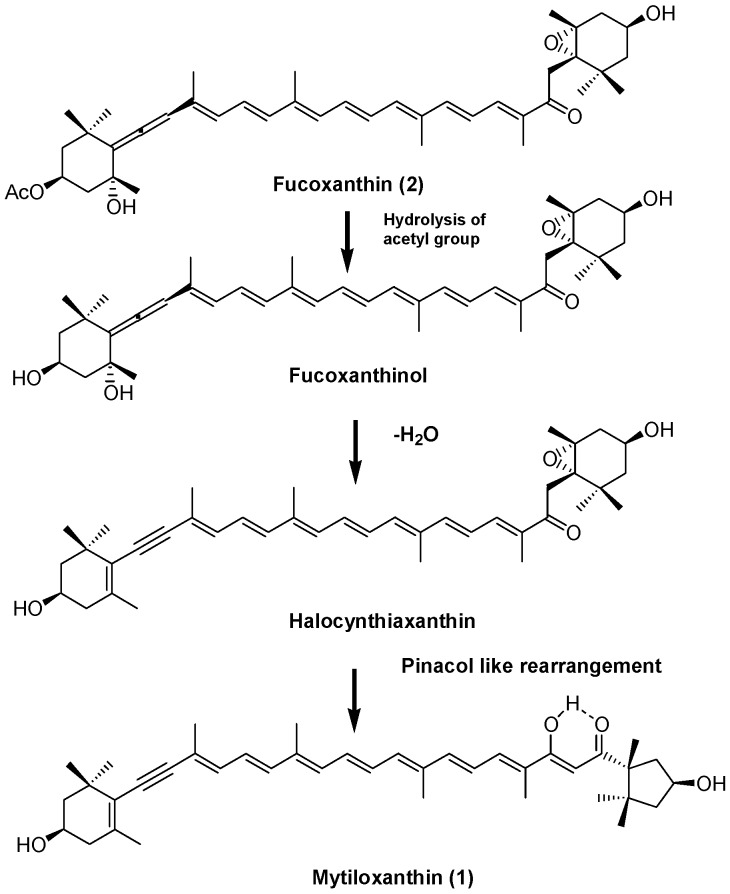
Metabolic conversion of fucoxanthin to mytiloxanthin in shellfish and tunicates.

It was reported that mytiloxanthin was converted from fucoxanthin (**2**) through a pinacol-like rearrangement [1,2,4]. Namely, dietal fucoxanthin (**2**) from diatoms was a metabolite for mytiloxanthin (**1**) via fucoxanthinol and halocynthiaxanthin in shellfish and tunicates, as shown in Figure 2 [1,2,9,10].

It is well known that marine carotenoids such as astaxanthin show excellent anti-oxidative activity [11,12]. However, because of limited availability from natural sources, the anti-oxidative activity of mytiloxanthin has not yet to be reported. Therefore, we synthesized mytiloxanthin and studied its quenching effect on singlet oxygen, its scavenging effect on hydroxyl radicals and its inhibitory effect on lipid peroxidation of mytiloxanthin. In the present paper, we present these experimental results.

## 2. Results and Discussion

In order to investigate the anti-oxidative activities of mytiloxanthin, it was synthesized using a recently reported method [8], as described in the Section 3 [8]. Furthermore, β-carotene, astaxanthin, and fucoxanthin were used as positive controls of the anti-oxidative activity.

**Figure 3:**
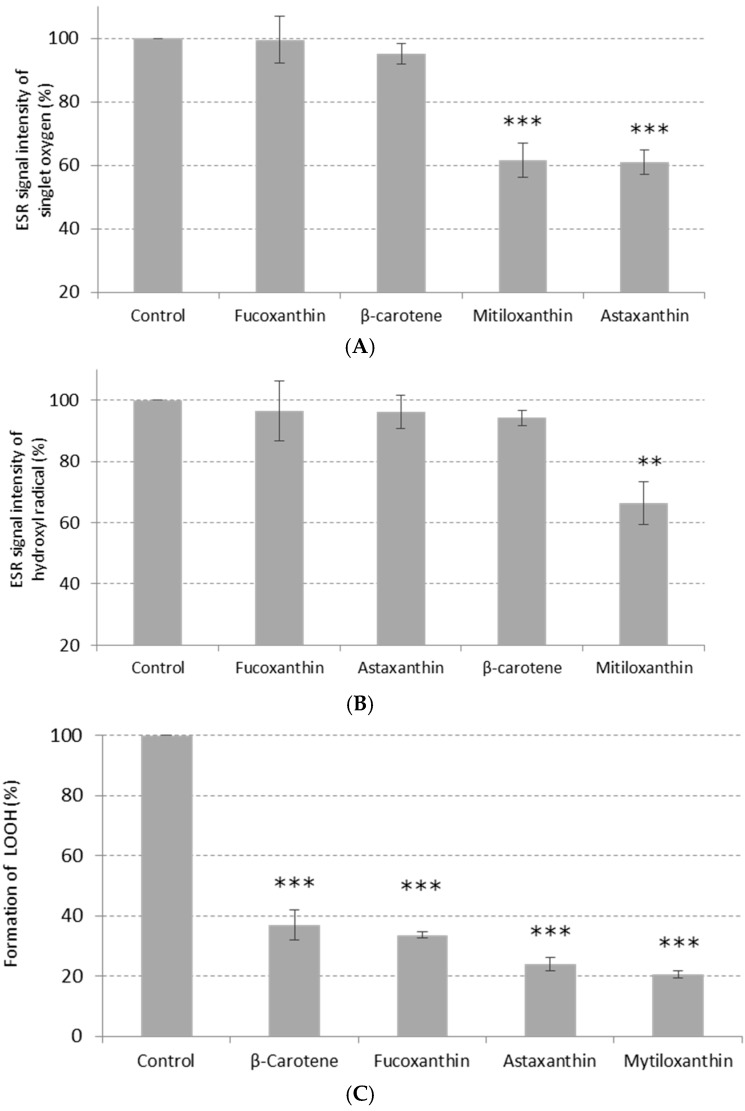
Scavenging activities (% of control group) for singlet oxygen (**A**) and hydroxyl radicals (**B**) and inhibitory effect (% of control group) on the lipid peroxidation (**C**) of carotenoids. Significance compared with the control group: * *p* < 0.05, ** *p* < 0.01, *** *p* < 0.01.

Figure 3A,B show the scavenging effect (% of control group) on singlet oxygen and hydroxyl radicals, respectively, by carotenoids. Mytiloxanthin showed almost the same quenching activity (61.6%) for singlet oxygen as that of astaxanthin (61.0%), which is a well-known and excellent singlet oxygen quencher [11,12]. On the other hand, fucoxanthin, a precursor of mytiloxanthin, hardly showed singlet oxygen-quenching activity (99.5%) in this experimental system. It was reported that the singlet oxygen-quenching activity of carotenoids depends on the number of conjugated double bonds, polyene chain structures, and functional groups [12,13]. Fucoxanthin contains nine conjugated double bonds including one carbonyl group and one allenic group in its molecule. On the other hand, mytiloxanthin has an 11-conjugated-double-bond polyene system, including one acetylenic and one carbonyl group in its molecule. Therefore, it was suggested that the strong quenching activity for singlet oxygen shown by mytiloxanthin was due to this long conjugated polyene system.

It has been consistent that carotenoids do not directly scavenge superoxide anions or hydroxyl radicals [14]. Recently, Hama *et al.* reported that astaxanthin could scavenge hydroxyl radicals in a liposome system [15]. In the present study, we also found that mytiloxanthin could scavenge hydroxyl radicals (66.4%) and that this activity was markedly higher than that of astaxanthin (96.1%) in this experimental system. Fucoxanthin hardly showed scavenging activity for hydroxyl radicals as shown in Figure 3B.

Inhibitory activities of mytiloxanthin and fucoxanthin on lipid peroxidation were monitored by measuring the accumulation of methyl linolate hydroperoxides during the incubation of methyl linolate with 2,2′-Azobis(2,4-dimethylvaleronitrile) (AMVN) as a radical initiator, with astaxanthin and β-carotene as positive controls. Figure 3C shows the results of inhibitory activity on lipid peroxidation by carotenoids at a concentration of 2 mM (final concentration of 167 μM). Mytiloxanthin showed slightly stronger activity than astaxanthin, which is a well-known antioxidant [11,12], and it also showed higher activity than fucoxanthin and β-carotene. Several investigators have reported that an increased number of conjugated double bonds in carotenoids, and the presence of functional groups such as carbonyl and hydroxyl groups in carotenoids, enhance their anti-oxidant effects [11,12,13]. Therefore, it was suggested that the strong inhibitory activity on lipid peroxidation shown by mytiloxanthin was due to the presence of the long conjugated polyene system described above. Mytiloxanthin has an enolic β-diketone group. Along with the 3-hydroxy-4-keto-β-end group in astaxanthin, this enolic β-diketone group may contribute to anti-oxidative activity. 

Many crustaceans oxidatively convert dietary β-carotene to astaxanthin [1,2]. The scallop and sea angel also oxidatively convert dietary diatoxanthin to pectenolone [2,16]. By these oxidative metabolic conversions, the anti-oxidative activities of dietary carotenoids are increased. Therefore, these marine animals metabolize dietary carotenoids to a more active anti-oxidative form and accumulate them in their bodies and gonads.

Similarly, shellfish and tunicates accumulate fucoxanthin from dietary algae and convert it to mytiloxanthin. By this conversion, the carotenoid changes color from orange to red and shows increased anti-oxidative activities, as described above. Mytiloxanthin in shellfish and tunicates may contribute to protection against oxidative stress and promote reproduction, similarly to astaxanthin in crustaceans and pectenolonein in the scallop and sea angel.

## 3. Experimental Section

### 3.1. Reagents

Hematoporphyrin, riboflavin, and hydrogen peroxide were purchased from Wako Pure Chemicals (Osaka, Japan); 2,2,6,6-Tetramethyl-4-piperidone (TMPD) was purchased from Aldrich (Milwaukee, WI, USA); 5,5-Dimethyl-1-pyrroline-*N*-oxide (DMPO) was purchased from Labotec (Tokyo, Japan); 2,2′-Azobis(2,4-dimethylvaleronitrile) (AMVN) as purchased from Wako Pure Chemicals (Osaka, Japan). Methyl linolate, β-carotene, and astaxanthin were purchased from Aldrich (Milwaukee, WI, USA). Fucoxanthin was prepared from brown algae. Mytiloxanthin was synthesized as described below. Figure 1 shows structures of carotenoids used in this study.

### 3.2. ESR Spin-Trapping Analysis

ESR spectra were recorded at room temperature on a JEOL JES-FR30 spectrometer (JEOL, Tokyo, Japan) using an aqueous quartz flat cell (Labotec, Tokyo, Japan). TMPD was used as a singlet oxygen-trapping agent, and DMPO was used as superoxide anion and hydroxyl radicals, respectively. Superoxide anion radical (O_2_^−^) and OH were generated by addition of both the 100 μL of 25 μM of riboflavin, or 100 μL of 8 mM H_2_O_2_ solution and 10 μL of 250 mM DMPO to 100 μL of 8.8 μg/mL carotenoid CH_3_CN solution by UV-A irradiation. In a similar manner described above, ^1^O_2_ was generated by the addition of both the 100 μL of 0.25 mM hematoporphyrin and 10 μL of 500 mM TMPD to 100 μL of 8.8 μg/mL of carotenoid CH_3_CN solution by UV-A irradiation. ESR spectra were started simultaneously to measure after UV-A irradiation. The all spin-trapped ESR spectra were monitored between the third and fourth signals from the low magnetic field due to the external standard, Mn(II)-doped MnO. 

### 3.3. Inhibition of Lipid Peroxidation

Carotenoids were dissolved in EtOH at a concentration of 2 mM (final concentration of 167 μM in the reaction mixture). The sample solution, 100 μL, was added to 1 mL of 100 mM methyl linolate solution [*n*-hexane/2-propanol (1:1, *v*/*v*)], and the solution was incubated at 37 °C for 5 min. As a control, EtOH alone was used instead of the sample solution. The oxidation reaction was then performed by adding 100 μL of 100 mM n-hexane solution of AMVN and the mixture was incubated with air at 37°C. At regular intervals, the oxidation reaction products, methyl linolate hydroperoxides, were quantified by high performance liquid chromatography (HPLC). HPLC was performed with a Hitachi L-6000 intelligent pump and an L-4250 UV-VIS detector. The following HPLC conditions were employed for the quantitative analysis of methyl linolate hydroperoxides: column Lichrosorb Si 100 (5 μm particle size) (4.6 × 250 mm) (Merck, Damstraat, Germany); solvent system: 2-propanol/n-hexane (1:99, *v*/*v*); flow rate: 1 mL/min; and detection: 235 nm.

### 3.4. Synthesis of Mytiloxanthin

**Scheme 1:**
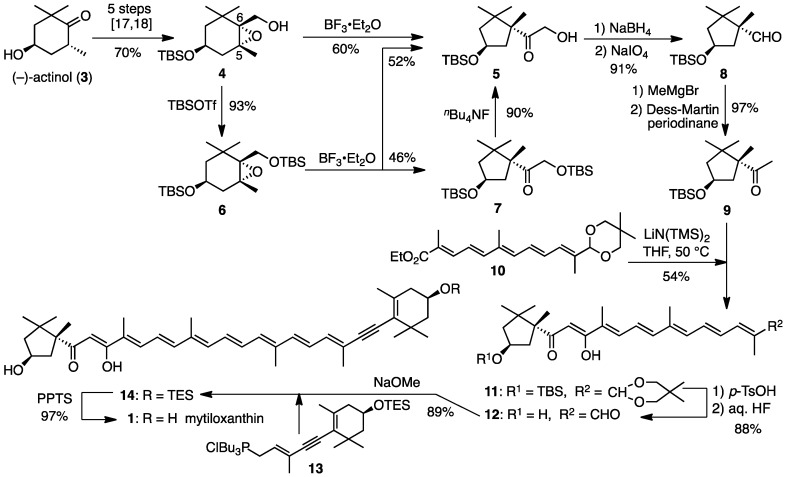
Synthesis of mytiloxanthin (**1**).

Mytiloxanthin (**1**) was synthesized by a recently reported method [8] as shown in Scheme 1. The anti(α)-epoxy alcohol **4**, stereoselectively prepared from (–)-actinol (**3**) [17,18], was treated with BF_3_·OEt_2_ to provide the cyclopentyl ketone **5** in 60% yield, by opening of C-6-oxygen bond of the oxirane ring and subsequent ring contraction. The yield of **5** from **4** was improved by a stepwise route through the di-*tert*-butyldimethylsilyl (TBS) ether **6** (86% for 3 steps). The compound **5** was converted into the methyl ketone **9** in four steps and this was condensed with the separately prepared conjugated ester **10** to give the desired β-diketone **11**. After hydrolysis of acetal moiety of **11** and subsequent desilylation, the resulting apocarotenal **12** [5] was condensed with the acetylenic phosphonium salt **13** [19] and then desilylated to preferentially provide all-*E*-mytiloxanthin (**1**) in a good yield. The total yield of **1** from epoxy alcohol **4** was 30% over 13 steps (21% from (–)-actinol over 18 steps).

## 4. Conclusions

The anti-oxidative activities of mytiloxanthin, a metabolite of fucoxanthin in shellfish and tunicates, were investigated. Mytiloxanthin showed excellent anti-oxidative activities for quenching singlet oxygen, scavenging hydroxyl radicals, and inhibiting lipid peroxidation. These activities were higher than those of fucoxanthin as a precursor of mytiloxanthin. Therefore, it was suggested that marine animals accumulate dietary fucoxanthin and convert it to a more anti-oxidative active form, mytiloxanthin.
